# Leaders’ Gender, Perceived Abusive Supervision and Health

**DOI:** 10.3389/fpsyg.2018.02427

**Published:** 2018-12-05

**Authors:** Christiane R. Stempel, Thomas Rigotti

**Affiliations:** ^1^Work and Organizational Psychology, Medical School Hamburg, Hamburg, Germany; ^2^Work, Organizational and Business Psychology, Johannes Gutenberg University Mainz, Mainz, Germany

**Keywords:** gender, abusive supervision, leadership, health, role congruity theory

## Abstract

**Purpose:** We investigated the role of gender in abusive leadership practices, along with the effects of abusive leadership on employee health. We tested two hypotheses regarding the relationship between abusive leadership practices and subordinates’ health outcomes.

**Design:** At two points of measurement, 663 participants in Germany rated their 158 direct team leaders on abusive supervision and stated their own levels of emotional exhaustion and somatic stress. To test our hypotheses, we used a mixed model approach.

**Findings:** The results show no gender differences between the ratings for female and male leaders regarding abusive supervision but do confirm that the leaders’ gender did play a role in employees’ perceptions of abuse; perceived abusive supervision is more strongly related to increased emotional exhaustion and somatic stress when the leader is male.

**Limitations:** The generalizability of the study is limited due to a majority of females in the sample.

**Practical Implications:** Organizations should review their policies and procedures to first identify abusive supervision, then to offer adequate support programs for both leaders and subordinates.

**Originality/Value:** The study integrates gender into research on leadership and health, shifting the focus from previous studies that investigated constructive to destructive leadership. A further strength of the study is the application of a multilevel design and two separate points of measurement.

## Introduction

This study addresses the question of whether the relationship between abusive supervision and subordinate health, differs depending on the gender of the supervisor. To investigate this issue, we first turn to leadership and health. Leadership can be regarded as a key factor in the well-being and health of subordinates (Kuoppala et al., 2008; Lyons and Schneider, 2009; Skakon et al., 2010; Mullen and Kelloway, 2011), particularly concerning destructive leadership behaviors, such as abusive supervision (Nyberg et al., 2011; Martinko et al., 2013; Schyns and Schilling, 2013). It is important to note that leadership is not unidirectional, but instead a bidirectional interaction between a leader and a subordinate (Van Dierendonck et al., 2004). Thus, perceptions of leadership may be as important as actual behavior (Schyns, 2007; Brees et al., 2016; Schyns et al., 2018). Here, gender comes into play. The gender of a leader is a salient characteristic that shapes the perceptions and attitudes of subordinates (e.g., Eagly and Karau, 2002; Parks-Stamm et al., 2008; Schieman and McMullen, 2008). As past research on constructive leadership has demonstrated, people perceive, evaluate and acknowledge similar behavior between female and male leaders differently (Eagly et al., 1992; Schein, 2001; Sczesny, 2003; Johnson et al., 2008; Kulich et al., 2011; Amanathulla and Tinsley, 2013). The consequences of these different perceptions are highly significant for female leaders, who must often overcome greater barriers to reach leadership positions (e.g., Schein, 2001), face harsher standards (Ryan and Haslam, 2007) and receive less acknowledgment, fewer rewards, and lower pay (cf. Kulich et al., 2007) than their male counterparts. The Role Congruity Theory suggests that, due to similar social norms of men and leaders, men are perceived as more legitimate in leadership positions (Eagly and Karau, 2002; Heilman and Haynes, 2005). However, what happens to role congruity when the leader is not a shining example and behaves destructively? In this study, we investigated how gendered perceptions of leaders, shape the relationship between abusive supervision and health.

This study extends previous research in several ways. Firstly, two different theoretical roots of the Role Congruity Theory were re-evaluated with respect to destructive leadership: the Stereotype Content Model (Fiske et al., 2002) and the Expectations State Theory (Ridgeway, 2001, 2011). Secondly, by shifting the focus to destructive leadership behaviors, we provide new insights on the interplay between leadership and gender. Thirdly, choosing health outcomes as a dependent variable is an asset of the study, revealing the impact leadership behavior has on the individual. Thus, unlike variables such as performance or effectiveness, our outcomes were not entangled with the actual tasks or achievements of the leader. Fourth, since the study integrated research on leadership and gender, Germany, which is considered a country emphasizing ambition and differentiated gender roles (Hofstede et al., 2010), provided an interesting research setting (Chhokar et al., 2013).

## Theoretical Background

### Leadership and Well-Being

Leadership has been identified as important for the well-being and health of employees (Kuoppala et al., 2008; Mullen and Kelloway, 2011; Nyberg et al., 2011; Holstad et al., 2013). Leaders can influence their subordinates’ health either directly, by exhibiting supportive or obstructive leadership behavior (Skakon et al., 2010; Schyns and Schilling, 2013), or indirectly by influencing employee duties (Nielsen et al., 2008; Tuckey et al., 2012).

Abusive supervision is defined as “subordinates’ perception of the extent to which supervisors engage in a sustained display of hostile verbal and non-verbal behaviors, excluding physical contact” (Tepper, 2000, p. 178) and has been linked to increased health impairment and reduced well-being (for a review, see Martinko et al., 2013). Abusive behavior of leaders over time (Tepper, 2007) is likely to cause strain. Abusive supervision correlates with subordinates’ depression, anxiety, and emotional exhaustion (Tepper, 2000; Wu and Hu, 2009), as well as with more negative attitudes toward the job, the organization, and life in general, higher turnover intentions, and work–family conflict (Tepper, 2000, 2007). In their meta-analysis, Schyns and Schilling (2013, p. 11) note that “the effect size for the relationship of destructive leadership with well-being (rho = -0.35) even exceed those for constructive leadership (rho = 0.26).”

In the current study, we employed emotional exhaustion as a psychological indicator (Maslach and Leiter, 2008) and somatic stress as a physical (Pejtersen et al., 2010) indicator of health impairment. Drawing on previous findings regarding abusive supervision, it was expected that the hostile behaviors of abusive supervisors would be linked to higher levels of emotional exhaustion and somatic stress among their subordinates. Thus, we propose that:

H1: Abusive supervision positively relates to subordinates’ emotional exhaustion.

H2: Abusive supervision positively relates to subordinates’ somatic stress.

Since abusive supervision, by definition, relies on subordinates’ perceptions (Tepper, 2000), this relationship is likely influenced by the characteristics of the leader. Gender, one of the most salient characteristics, not only shapes perceptions but also affects the impact of leadership behavior (Eagly and Karau, 2002; Mohr and Wolfram, 2008; Parks-Stamm et al., 2008; Ye et al., 2016). To what extent behavior is seen as inappropriate or unjust differs for female and male leaders (Heilman and Eagly, 2008; Escartín et al., 2011; Amanathulla and Tinsley, 2013) and thus might cause differing health responses (Hoel et al., 2004). In the following section, we will therefore examine the role of gender, in the context of leadership and how it might shape the effects of abusive leadership on employee health.

### Theories on Leadership and Gender

Most existing research has focused on desirable leadership behaviors. Prior research on gender, in the context of leadership, was built on three theories: the Stereotype Content Model (Fiske et al., 2002); the Expectations State Theory (Ridgeway, 2011); and the Role Congruity Theory (Eagly and Karau, 2002). All three theories are strongly interrelated but vary in their emphasis and scope.

The Stereotype Content Model concentrates on attributes ascribed to social groups (e.g., women, men, ethnic groups), which can be arranged into two dimensions; warmth and competence. For instance, the female gender stereotype would suggest that people rate women high on positive intentions (warmth) but low on agentic abilities (competence), whereas the opposite categorization would apply for men. Studies show that competence closely relates to status and lack of warmth closely relates to competition (Fiske et al., 2002). Both attributes are considered essential for leadership (Schein, 2001). The theory describes stereotypes of specific groups and respective reactions well, but sometimes falls short on explaining why reactions differ for individuals of social groups that fall into “conflicting” categories. For instance, a study by Cuddy et al. (2004) shows that working mothers are perceived less competent, whereas working fathers retain their competence and gain on perceived warmth. Thus, keeping in mind that stereotypes are also subject to change (Duehr and Bono, 2006), it seems necessary to take further factors into consideration.

The Expectations State Theory (Ridgeway, 2001, 2011) emphasizes beliefs about status that are associated with a variety of social roles in society. People in high-status roles are ascribed higher competence, as well as more valuable skills and emerge as leaders more often. Due to an institutionalized social hierarchy, men are considered the higher- and women the lower-status group. These implicit expectations drive the self’s, as well as others’, performance expectations.

The Role Congruity Theory (Eagly and Karau, 2002) integrates both aforementioned theories by incorporating social roles as well as social groups. The representation of social groups in occupational roles (e.g., male leaders) leads people to associate group stereotypes with the occupational role (Koenig and Eagly, 2014). Yet, it is important to note that role congruity can occur on two levels: first, behaviors and attributes ascribed to the gender and the leader role can coincide. For instance, competence is seen as necessary for leadership and more likely ascribed to men (Stereotype Content Model). Second, the higher positional status (e.g., leader) can match the higher gender status as in the case of men (Expectations State Theory).

Regarding gender and leadership, the male leaders’ social roles align with their workplace leadership roles in both cases, while those of women do not (Schein et al., 1996; Schein, 2001; Koenig et al., 2011). This ‘think manager, think male’ phenomenon (TMTM; Schein, 2001) has been reported in many studies from various countries (Willemsen, 2002; Garcia-Retamero and López-Zafra, 2006; Ryan et al., 2011).

With respect to constructive leadership behaviors, there seems to be a preference for the stereotype content aspect of the Role Congruity Theory (e.g., Eagly and Carli, 2003; Sczesny, 2003; Heilman et al., 2004). Because of stereotype incongruence, women face barriers, since they are not seen as competent enough or are considered too communal for leadership (Heilman and Haynes, 2005). However, this focus on stereotype incongruence neither takes into account potential changes of stereotypes (Duehr and Bono, 2006; López-Zafra and García-Retamero, 2012), nor does it explain several mixed findings (Embry et al., 2008; Koenig et al., 2011; Paustian-Underdahl et al., 2014).

For instance, contrary to the TMTM phenomenon, women show transformational leadership behaviors more often and these behaviors are perceived as being more stereotypical of female leaders (Eagly et al., 2003; Eagly, 2007; Stempel et al., 2015). Nevertheless, a study by Mohr and Wolfram (2008) demonstrated that leaders’ considerate verbal behaviors impact subordinates’ health more when enacted by male leaders, as opposed to female leaders. These findings suggest that even when women show desirable leadership behaviors, they might still not be as influential as male leaders. Here, the Expectations State Theory argues that the established social role of the leader is associated with men, and therefore their leadership behaviors – desirable or not – are more weighted and influential than those of female leaders (Embry et al., 2008; Ridgeway, 2011).

#### Theoretical Perspectives on Gender and Destructive Leadership

Few studies have investigated the role of gender in the context of less constructive, or destructive, leadership (Eagly et al., 2003; Lee and Brotheridge, 2011; Wang et al., 2013). When the Role Congruity Theory is applied to desirable leadership behavior, a differentiation between stereotype content (Stereotype Content Model) and social roles (Expectations State Theory) is usually not required, because men benefit from the congruence of the higher-status leader role as well as associated gender stereotypes. Nevertheless, blurring the two aspects might not only account for mixed results (Mohr and Wolfram, 2008; Wolfram and Mohr, 2010), but also makes it difficult to derive predictions about role congruity, with respect to abusive supervision. Depending on the weighting of the aspects of the Stereotype Content Model and the Expectations State Theory, the Role Congruity Theory allows for opposing predictions regarding the moderating effect of the leaders gender on subordinates’ health. Since there is, to our knowledge, no clear empirical evidence on the subject, in the context of abusive supervision, the following two sections provide argumentation for two competing hypotheses.

According to the Stereotype Content Model, abusive supervision behavior might damage the competence, as well as the warmth ratings attached to the leader stereotype. It remains unclear, however, how reactions to the stereotype of a ‘bad male leader,’ as opposed to a ‘bad female leader,’ would differ, since both would score low on warmth and competence. We can only make predictions regarding the consequences of abusive supervision for subordinate health, when taking the Role Congruity Theory’s perspective on stereotype content into account. Assuming that assertive and aggressive behavior is more in line with the male gender stereotype (Schein, 2001), abusive supervision by a male leader would speak for role congruence. This overlap between the male gender and the leader role might make the behavior more predictable and acceptable and therefore, may cause less harm to subordinates health. Accordingly, for abusive female leaders, the violation of communal gender stereotypes would cause more negative judgments, due to their incongruence, and subordinates might feel stressed, because the behavior was less expected and predictable. Thus, we propose that the perceived behavioral incongruence for female leaders strengthens the relation between abusive supervision and health outcomes:

H3: A leader’s gender moderates the relationship between abusive supervision and (a) subordinates’ emotional exhaustion and (b) somatic stress such that the relationship is stronger for female than for male leaders.

Emphasizing the Expectations State Theory aspect of the Role Congruity Theory, allows for differing predictions regarding the consequences of abusive supervision on subordinate health. According to the Expectations State Theory, men are the higher-status group and are considered more legitimate and influential in the leadership role. Consequently, their actions carry considerably more weight, making them more essential in the eyes of their subordinates, and thus might have a stronger impact on their health (Embry et al., 2008; Mohr and Wolfram, 2008; Ridgeway, 2011). Accordingly, for female leaders, the violation of role norms would make them appear even less congruent and legitimate for a leadership position; therefore, subordinates would ascribe female leaders lower levels of power to influence their well-being. Even though these assumptions are contrary to those outlined in the previous section, this reasoning is equally in line with the Role Congruity Theory, since there is congruence between the leader and the gender role for men but not for women (Schein, 2001). Thus, we propose that the perceived status incongruence for female leaders, buffers the relationship between abusive supervision and health outcomes:

H4: The leaders’ gender moderates the relationship between abusive supervision and (a) the subordinates’ emotional exhaustion and (b) somatic stress such that the relationship is stronger for male than for female leaders.

## Materials and Methods

### Participants and Procedure

The sample for this study is part of an international research project on leadership and well-being (Rigotti et al., 2014). The survey was carried out in accordance with the recommendations of the Federation of German Psychologists Association’s Code of Ethics. We conducted the study with the permission of the workers’ council of the participating organization, and we informed the participants about the objectives and procedures. Participation in the study was voluntary, and confidential handling of the data was assured. Thus, the procedures of the survey are in accordance with the Declaration of Helsinki. Ethical review and approval were not required for this study as per the institutional and national requirements.

A precondition for participation in the study was that the participants have frequent contact (usually on a daily, but at least weekly, basis) with their immediate supervisors. In Germany, 2,567 subordinates from nine different companies (banks, medium-sized enterprises, public sector) were invited to take part in the first data collection in 2012, and 1,594 individuals participated, resulting in a response rate of 62.1%. Participants rated their direct supervisors on abusive supervision. Six months later, 2,093 subordinates were invited and 1,120 responded (response rate of 53.5%), rating their well-being in terms of emotional exhaustion and somatic symptoms. The survey followed a two-wave multilevel design. We identified subordinates by an individual code and matched them to their respective leaders by a specific team code. Participants were provided with the option to complete the questionnaire online, via a link in an email, or a paper-pencil version, with the majority (97.9%) opting for the online choice. Drop-out analysis on all employed variables, only revealed that participants who dropped out of the sample at T2 were more likely to be male (*χ*^2^ = 8.41, *p* = 0.005) and have a permanent contract (*χ*^2^ = 6.05, *p* = 0.018); however, we excluded employees with temporary employment contracts (*n* = 38) as they might hold different expectations of leadership (cf. De Cuyper et al., 2008). Missing values further reduced the sample for analysis. We obtained a final sample of 663 subordinates who rated the behavior of their 158 immediate supervisors. Thus, on average, four team-members responded per team.

In the final two-wave sample, 62.9% of the leaders were female and 37.1% were male. The participants were, on average, 41.4 years old (*SD* = 9.48), and 80.8% of the respondents were female. On average, subordinates had worked for their current leaders for about 5 years (*M* = 4.87, *SD* = 4.04) and for their employers for approximately 15 years (*M* = 15.30, *SD* = 8.91). Participants worked an average of 38.5 (*SD* = 5.24) hours per week.

### Measures

In order to reduce same method bias, abusive supervision was measured at the first point of data collection, whereas the dependent variables of emotional exhaustion and somatic stress were measured with a time lag of 6 months.

#### Abusive Supervision

We assessed abusive supervision with a five-item scale, developed by Mitchell and Ambrose (2007). The subordinates indicated, on a seven-point Likert-type scale, how much they agreed with statements such as “My boss puts me down in front of others” (1 = strongly disagree to 7 = strongly agree; α = 0.93). Due to the limitations of Cronbach’s alpha (α = 0.93, McNeish, 2017), we also calculated the omega total coefficient (ω = 0.89). Factor loadings and residual variances were obtained from a multilevel confirmatory factor analysis.

#### Emotional Exhaustion

We evaluated emotional exhaustion using the three most prototypical items (De Cuyper et al., 2012) from the Maslach Burnout Inventory (Maslach et al., 1996). Participants were asked to indicate on a seven-point scale (0 = never to 6 = every day) how often a certain feeling occurred. A sample item is “I feel emotionally drained from my work.” The alpha value and total omega for the scale were both 0.85.

#### Somatic Stress

We applied a four-item measure from the COPSOQ II by Pejtersen et al. (2010) to capture symptoms of somatic stress. Participants were asked to indicate how often they felt “stomach aches,” “headaches,” “palpitations,” or “tension in various muscles” during the last 4 weeks (1 = not at all to 5 = all the time). The period of 4 weeks took into account that psychophysiological responses are more likely to become consolidated over a longer period of stressful events. According to Levenson (2011, p. 603) “for symptoms in general, 75% of the patients presenting to primary care improve within 2–4 weeks (Kroenke, 2003). Thus, it makes sense to rely on an initial 4–6-weeks wait to clarify whether the symptoms will persist” (Kroenke and Jackson, 1998). Because symptoms cannot be expected to occur simultaneously, a medium high α of 0.65 and an omega of 0.62 are acceptable for this index scale.

#### Gender

The gender of the participants and their respective supervisors was coded 0 for female and 1 for male. The supervisors reported their own gender, and we were also able to fill in missing values with information provided by the participating companies.

#### Controls

In testing our hypotheses, we used the following controls for subordinates: age, gender and tenure with the leader. Age and gender were included as a control variable in the analysis because they were shown to be relevant for well-being and health at work (Mauno et al., 2013), as well as for attitudes toward leaders (Wolfram et al., 2007). Age (years) was reported by the participants at the initial assessment, as was gender. Because effects of leadership are likely to depend on time spent working with the leader, we asked “How long have you been working under the supervision of this leader?” We measured this time period in years.

## Results

Table 1 shows descriptive statistics and correlations of our study variables.

**Table 1:** Means, SD, and intercorrelations among studied variables.

	M	*SD*	1	2	3	4	5	6	7
1 Leader gender^a^	0.37	0.48	-						
2 Subordinate gender^a^	0.19	0.39	0.13^∗∗^	-					
3 Age	39.90	9.84	-0.01	-0.11^∗∗^	-				
4 Tenure with leader	4.12	4.00	-0.07	-0.05	0.22^∗∗^	-			
5 Abusive supervision	1.33	0.76	-0.02	-0.04	0.07	-0.00	(0.93)		
6 Emotional exhaustion	2.68	1.36	-0.12^∗∗^	-0.00	0.01	-0.04	0.25^∗∗^	(0.85)	
7 Somatic stress	1.91	0.64	-0.03	-0.00	-0.00	-0.06	0.16^∗∗^	0.52^∗∗^	(0.65)


**M*, means; *SD*, standard deviation; ^*a*^gender was coded ‘0’ for women and ‘1’ for men; Cronbach’s alphas in parentheses; ^∗∗^*p* < 0.01.*

Since the data structure was nested (individuals within teams), multivariate variance analyses, using a team as a factor, were conducted. Results indicated a substantial variation across teams in exhaustion [*F*(158, 549) = 1.62, *p* < 0.001] and somatic stress [*F*(156, 532) = 1.50, *p* < 0.001]. We analyzed the null-model for all variables in order to estimate the ICCs. The ICC for the dependent variables exhaustion was *ICC(1)* = 10.2% and for somatic stress *ICC(1)* = 7.0%. These analyses confirmed that there was a considerable proportion of variance due to team membership in the dependent variables. Because we did not aggregate data, we deemed these results to indicate that a multilevel approach was appropriate (Bliese, 2000).

In the subsequent models, the dichotomous variables, gender of the subordinate (level 1) and gender of the leader (level 2), were included uncentered (Dawson, 2014). Abusive supervision, as well as the control variables of age and tenure with the leader (level 1), were entered grand-mean centered (Aguinis et al., 2013). Following suggestions from Aguinis et al. (2013), we adopted a stepwise procedure. We first tested main effect models comparing a random intercept model with a random slope model (including random slopes for abusive supervision). As the random slope model did not show a significantly better fit (*Δ-2LL* = 0.67, *Δdf* = 4, *p* = 0.48, one-tailed), the cross-level interaction model was tested with fixed slopes for abusive supervision.

### Hypotheses H1 and H2: Abusive Supervision and Health

Consistent with predictions, and as displayed in Table 2, the models including the main effects only show a significant relationship between abusive supervision and emotional exhaustion (ϒ_*model*1_ = 0.46, *p* < 0.001) and somatic stress (ϒ_*model*1_ = 0.11, *p* < 0.01).

**Table 2:** Hierarchical linear modeling results for emotional exhaustion and somatic stress.

		Emotional exhaustion			Somatic stress		
			
		Model 1 random intercept	Model 2 random slope	Model 3 cross-level-interaction	Model 1 random intercept	Model 2 random slope	Model 3 cross-level-interaction
Level 1	Intercept, *γ_*00*_*	2.65 (0.08)^∗∗∗^	2.65 (0.08)^∗∗∗^	2.65 (0.08)^∗∗∗^	1.91 (0.04)^∗∗∗^	1.91 (0.04)^∗∗∗^	1.91 (0.04)^∗∗∗^
	Subordinate gender *γ_*10*_*	0.08 (0.13)	0.08 (0.13)	0.07 (0.13)	-0.20 (0.06)^∗∗^	-0.19 (0.06)^∗∗^	-0.20 (0.06)^∗∗^
	Subordinate age *γ_*20*_*	0.00 (0.01)	0.00 (0.01)	0.00 (0.01)	0.00 (0.00)	0.00 (0.00)	-0.00 (0.00)
	Tenure with leader *γ_*30*_*	-0.01 (0.01)	-0.01 (0.01)	-0.00 (0.01)	-0.00 (0.01)	-0.00 (0.01)	-0.00 (0.01)
	Abusive supervision (A) *γ_*40*_*	0.46 (0.08)^∗∗∗^	0.46 (0.08)^∗∗∗^	0.35 (0.09)^∗∗∗^	0.11 (0.04)^∗∗^	0.13 (0.05)^∗∗^	0.06 (0.04)
Level 2	Leader gender (B) *γ_*01*_*	-0.33 (0.13)^∗∗^	-0.33 (0.14)^∗^	-0.33 (0.13)^∗^	-0.03 (0.06)	-0.03 (0.06)	-0.03 (0.06)
	*Cross-level interaction*						
	A × B *γ_*41*_*			0.36 (0.16)^∗^			0.16 (0.08)^∗^
	Variance components						
	Within-team (L1) variance	1.55 (0.10)^∗∗∗^	1.55 (0.10)^∗∗∗^	1.54 (0.10)^∗∗∗^	0.36 (0.02)^∗∗∗^	0.36 (0.02)^∗∗∗^	0.36 (0.02)^∗∗∗^
	Intercept (L2) variance	0.18 (0.07)^∗∗^	0.18 (0.07)^∗^	0.18 (0.07)^∗∗^	0.03 (0.01)	0.03 (0.01)	0.03 (0.01)
	Slope (L2) variance		0.01 (0.10)			0.01 (0.02)	
	Intercept-slope (L2) covariance		-0.01 (0.08)			0.00 (0.01)	
	-2 log likelihood	3218.56	3217.89	3212.52			
	Number of free parameters	18	22	20			


*N_*level 1*_ = 663; N_*level 2*_ = 158. *SE*, standard error. ^∗^*p* < 0.05, ^∗∗^*p* < 0.01, and ^∗∗∗^*p* < 0.001. The random slope model did not significantly differ from the random intercept model (*Δ-2LL* = 0.67, *Δdf* = 4, *p* = 0.48, one-tailed), therefore the cross-level interaction model was calculated with fixed slopes; The cross-level interaction model showed a significant improved fit to the random intercept model (*Δ-2LL* = 5.37, *Δdf* = 2, *p* = 0.03, one-tailed). As models were tested simultaneously with both dependent variables, – 2LL values did not differ for emotional exhaustion vs. somatic stress.*

### Hypotheses H3 and H4: Gender of the Leader as Moderator

**FIGURE 1:**
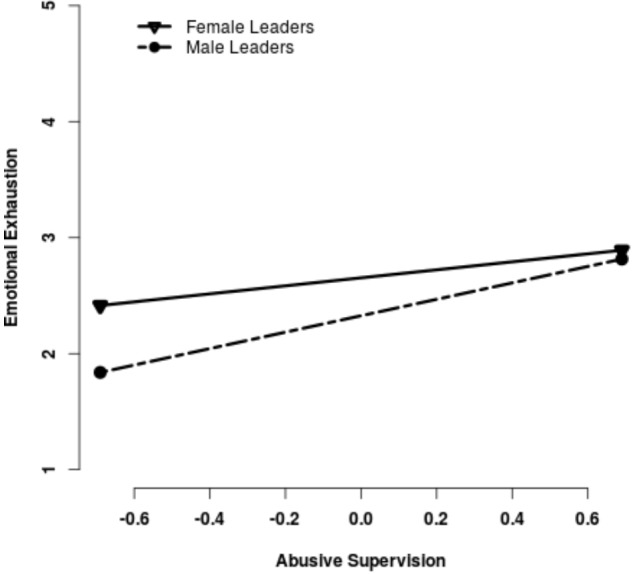
Slopes for the relationship between Abusive Supervision and Emotional Exhaustion.

**FIGURE 2:**
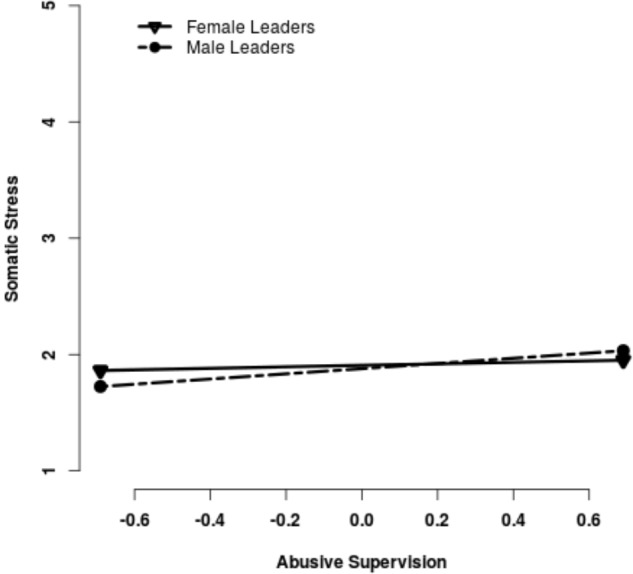
Slopes for the relationship between Abusive Supervision and Somatic Stress.

As predicted by H3 and H4 (see Table 2), the gender of the leader was found to be a significant moderator for emotional exhaustion (ϒ_*model*3_ = 0.36, *p* = 0.026) and somatic stress (ϒ_*model*3_ = 0.16, *p* = 0.039). With respect to the competing hypotheses, results indicated a stronger relationship for abusive male leaders than for abusive female leaders, as proposed in H4a and H4b (Figures 1, 2).

Simple slope analyses for the relationship between abusive supervision and emotional exhaustion showed significant slopes for female leaders (gradient 0.35, *p* < 0.001) and male leaders (gradient = 0.71, *p* < 0.001). For somatic stress, only the slope for male leaders was significant (gradient = 0.22, *p* = 0.013), indicating a positive relationship between abusive male supervision and somatic stress. No significant relationship emerged for female leaders (gradient 0.06, *p* = 0.146).

## Discussion

The current study investigated the role of gender in abusive leadership and the health of subordinates. As a first step, we showed that abusive supervision is linked to higher emotional exhaustion and more somatic stress. In a second step, the focus was on the role of the leaders’ gender. Even though participants did not report gender differences in the perception of abusive supervision, leader gender moderated the relationship between abusive supervision and health. The study demonstrated that the same behavior enacted by male and female leaders was associated with different health outcomes among their subordinates. Abusive male and female supervisors had subordinates reporting increased emotional exhaustion, but this relationship was considerably stronger for male leaders. In cases of somatic stress, the differences for female and male leaders were even stronger, since only abusive male supervisors had a significant relationship to somatic stress.

With two differentially focused hypotheses, the study explored whether status (Expectations State Theory, Ridgeway, 2011) or stereotype (in-)congruence (Social Content Model, Fiske et al., 2002) were critical in making predictions about abusive supervision and health. The findings offer a fresh look on the theoretical underpinnings of the Role Congruity Theory, revealing that it appears necessary to differentiate between the two aspects, particularly concerning destructive leadership. The current results support the notion that status congruence (male leader prototype) outweighs stereotypical behaviors (e.g., violating communal/agentic norms). Male leaders – even when exhibiting abusive supervision – seem to hold greater influence over their subordinates’ health.

The fact that participants rated the actual behaviors of their immediate supervisors, might explain this stronger emphasis on role norms. Stereotypes are usually applied in situations where people lack information about the target (Heilman and Eagly, 2008; Rosette and Tost, 2010). Thus, when confronted with abusive supervisors, people seem to rely more on role than on stereotype content information (Koenig and Eagly, 2014). This explanation would be in line with empirical findings regarding constructive leadership, showing that differences between actual female and male leaders are often considered small and context dependent (Vecchio, 2002; Paustian-Underdahl et al., 2014). The fact that similar behavior of female and male leaders still produces different effects on subordinate outcomes, has been demonstrated in various studies (e.g., Heilman and Haynes, 2005; Wolfram and Mohr, 2010; Kulich et al., 2011). Therefore, it seems necessary to take the status aspects of Role Congruity Theory into account when making predictions about the role of gender in abusive supervision, because it seems that people still ascribe male leaders more influence and legitimacy than female leaders.

It is important to note that the outcome variable in this study is the health of the subordinate. For other outcome variables like performance or sympathy, stereotype content congruence might be more pronounced. Here, subordinates might judge female leaders more harshly for violating their role as well as gender expectations. However, the power to influence seems to be associated with the leader role, which is more readily ascribed to men (Kulich et al., 2011).

Another condition may be the cultural setting. According to Hofstede et al. (2010), Germany scores 66 on a scale from 1 to 100 on masculinity and is, therefore, considered a country with masculine norms when compared to other countries. Leaders are expected to be decisive and assertive. These rather agentic role expectations point to a strong male-leader prototype, which might enhance the status aspect of the leader role, irrespective of the actual leadership behaviors.

Furthermore, since abusive supervision depends, by definition, on the perceptions of the subordinates, researchers should take attributional processes into consideration (Martinko et al., 2012; Burton et al., 2014). In a recent study, Schyns and colleagues (2018) highlight the importance of perceptions and attributions regarding abusive supervision. But to date, we have no knowledge of studies dealing with gendered attributions of abusive supervision, even though several researchers acknowledge that gender (Hoel et al., 2004; Burton et al., 2014; Salin, 2015), as well as status and power differences, might be of importance (Escartín et al., 2011; Samnani et al., 2013).

Overall, this study offers two theoretical perspectives on the Role Congruity Theory in the context of abusive supervision and gender. Differentiating between the social role itself and associated gender stereotypes, may help to explain some of the mixed results in studies on gender and leadership. Nevertheless, additional research is necessary to clarify whether the theoretical assumptions made in this study, hold empirically across differing contexts.

### Limitations, Strengths and Directions for Future Research

Since this study addresses leader gender as a moderator, the predominance of female participants in our sample was somewhat problematic. Effects for male leaders were significant, despite them being the minority in the sample. Nevertheless, as it has been shown that perceived gender roles differ across branches and teams (Embry et al., 2008; Koenig et al., 2011; Korek et al., 2014; Paustian-Underdahl et al., 2014), future studies should take the organizational context into account.

Additionally, the floor effects of the abusive supervision variable might be problematic for the robustness of the statistical analysis. Abusive supervision seems to be a low-frequency phenomenon that nevertheless has a strong effect on health and well-being outcomes. Another potential limitation to consider, is the time frame used in this study. For separation of the independent and dependent variables, a time lag of 6 months was chosen in order to avoid common method bias, however, little is known about the time it takes for the detrimental effects of abusive supervision to appear or how long it takes for health outcomes to manifest.

Furthermore, because we expected that perceptions would play a significant role in investigating the proposed research question (Schyns et al., 2018), all independent and dependent variables were rated from the perspective of the subordinates. Brees et al. (2016) showed that subordinates’ characteristics were an important factor when it came to the perception of abusive supervision. Our study controlled for gender of the subordinate, and the nested data structure was taken into account, ensuring that every subordinate rated his or her immediate team leader.

As the findings suggest, it is important to consider leader gender as a moderator for abusive supervision and its effects on employee health. Even if the results reported here can help to explain the mixed results of previous studies, additional theoretical and empirical work is necessary to investigate the assumptions about the two different key aspects of the Role Congruity Theory. It needs to be clarified how distinct social role status is from stereotype content, how they interrelate and under what conditions one or the other might reveal its strength. For example, researchers could focus on changes in gender role norms over time. As mentioned with respect to stereotype content, agentic behaviors are shown to be increasingly associated with women (Diekman and Eagly, 2000; Duehr and Bono, 2006; Embry et al., 2008). Thus, it would be interesting to investigate how these changing gender stereotypes might affect the status perceptions of social roles.

The current study focused on leadership and negative indicators of health. Prospective studies should also take positive health and well-being indicators, such as general health or job satisfaction, into account. Since there is very little research on gender as a moderator in the relationship between abusive leadership and various organizational outcomes, it would also be interesting to extend this research question to other outcomes, such as performance, rewards or attitudes.

### Practical Implications

Regardless of gender, it seems important to identify leaders who exert abusive supervision in order to prevent considerable damage to subordinates’ health. For this reason, organizations should review their current policies and personnel procedures to recognize factors (Tepper et al., 2011; Neves, 2014) that contribute to the occurrence of abusive supervision and offer adequate programs to train supervisors (Gonzalez-Morales et al., 2016).

Organizations should raise gender consciousness in order to improve their corporate climate. Implementing a gender-sensitive approach to training programs along with guidelines for leaders which could help to strengthen constructive leadership (Kelloway and Barling, 2010) and make the work of female leaders more visible. Since the results suggest that males are, indeed, perceived as more legitimate in leadership positions, human resource professionals should take this into account during selection processes and performance evaluations. Female leaders need to be rewarded for good and health-oriented leadership performance in the same way their male counterparts are but should likewise be held responsible when showing poor performance.

## Conclusion

As the results demonstrate, the gender of the leader is important to consider when studying abusive leadership and its effects on health. We showed that abusive male leaders seem to have a more significant influence on their subordinates’ health than their female colleagues do. Taking a closer look at the theoretical implications of the Role Congruity Theory (Eagly and Karau, 2002), it seems necessary to differentiate between the status of social roles and the stereotype content. Underscoring the status aspect, the results of this study suggest that men are indeed perceived as more legitimate in their leadership positions, even when they exert detrimental leadership behaviors. If and how this gender difference translates into differential consequences in terms of evaluation, career prospects or well-being for female and male leaders, is an unexplored question that opens a broad avenue for future studies.

## Ethics Statement

We conducted this study in accordance with the ethical standards of the Institutional and National Guidelines and with the Declaration of the World Medical Association (2013). We informed all participants of the objectives and the procedures prior to initiating the study. In the form of oral presentations, the participants were informed that participation was voluntary, and the data was treated anonymously. We obtained informed consent from all individual participants by virtue of survey completion. Ethical review and approval were not required for this study as per the institutional and national requirements.

## Author Contributions

All authors worked together in a joint research project on leadership and health. Discussions and feedback processes happened on a regular basis. CS contributed to the general research question, the theoretical underpinning, the hypotheses and the discussion and wrote major parts of this manuscript. TR contributed to the methodological approach and the presentation of the results and reviewed the other sections and provided feedback.

## Conflict of Interest Statement

The authors declare that the research was conducted in the absence of any commercial or financial relationships that could be construed as a potential conflict of interest.
